# The use of non-linear tools to analyze the variability of force production as an index of fatigue: A systematic review

**DOI:** 10.3389/fphys.2022.1074652

**Published:** 2022-12-14

**Authors:** Fernando García-Aguilar, Carla Caballero, Rafael Sabido, Francisco J. Moreno

**Affiliations:** Sport Sciences Department, Miguel Hernández University of Elche, Elche, Spain

**Keywords:** force control, strength, non-linear, complexity, neuromuscular fatigue

## Abstract

**Background:** Fatigue is a process that results in a decreased ability to produce force, and which could eventually affect performance and increase the risk of injury. Force variability analysis has been proposed to describe the level of fatigue with the purpose of detecting the development of fatigue. Variability is credited to play a functional and adaptive role through which the components of a system self-organize to solve a motor problem. Non-linear tools have been applied to analyze the variability of physiological signals, revealing that the structure of motor fluctuations provides relevant information about the functional role of variability. It has been suggested that the presence of lower complexity in the variability structure could reveal a less functional and adaptative state (e.g., ageing or illness). In the last years, an increased number of studies have applied these techniques to force variability analysis in relation to fatigue.

**Objective:** To provide an overview of the current knowledge on the use of non-linear tools on force variability as a fatigue index.

**Methods:** Following PRISMA guidelines, a systematic search of SPORTDiscus, Scopus, Web of Science and PubMed was carried out. Studies included were: a) original studies that analyzed the effect of fatigue on humans during an action focused on force production; b) published studies with their title and abstract in English; c) studies that applied non-linear tools on a signal directly related to force production.

**Results:** Twenty-five studies were included in this review. The relationship between fatigue and the complexity of force variability, the type of action and relative intensity, the nature of the signal and the non-linear tools used, and the methods of data acquisition and processing were identified.

**Conclusion:** The articles reviewed suggest that fatigue leads to a decrease in complexity mostly in isometric contractions, but this is not as clear in dynamic contractions. This fatigue-induced loss of complexity seems to be a result of changes in the nervous system at the central level, albeit triggered by peripheral mechanisms. It should be noted that non-linear tools are affected by the relative intensity of contraction, non-stationarity, and the acquisition and treatment of the signal.

## Introduction

Fatigue can be defined as a multifactorial process resulting in a decrease or failure to produce force by a muscle or muscle group (Gandevia, 2001; Enoka and Duchateau, 2008; Carroll et al., 2017). This decrease in force production affects the ability to perform a motor task (Taylor and Gandevia, 2008; Ament and Verkerke, 2009; Boyas and Guével, 2011; Carroll et al., 2017), reducing contractile function and muscle activation (Enoka and Duchateau, 2016), and it is related to an increase in the risk of injury (Almonroeder et al., 2020). The onset of fatigue is an intricate process that affects the organism in different ways depending on factors which may be either intrinsic (e.g., age, sex, body composition) or extrinsic (e.g., features of the task, environmental conditions) (Enoka and Duchateau, 2008). These factors impact on the development of fatigue (central or peripheral) and how it reveals itself (Place and Millet, 2020), and, thus, on how we can detect fatigue in the organism.

Classical measures of fatigue, such as maximal voluntary contraction force (MVC) or energy output, may be an index of fatigue, but they do not provide information on the intensity of fatigue (Enoka and Duchateau, 2016), i.e., they do not allow us to quantify the fatigue state of the organism. In addition, different methods are recommended depending on both the task and the predominant source of fatigue (Zwarts et al., 2008; Place and Millet, 2020). Among the methods used to examine the level of fatigue in humans, the variability in force production has been proposed in several studies (Slifkin and Newell, 2000; Contessa et al., 2009; Cortes et al., 2014). Motor variability is considered to be the variations or fluctuations that occur in motor behavior during the repetitive execution of an action (Stergiou, 2004). The production of muscle force involves multiple interacting elements (e.g., motor neurons, myofibrils, tendon units) (Badillo, 2002), which vary along different time scales (e.g., nerve impulses vary over smaller time scales than joint movement variations). Therefore, it can be assumed that these variations represent how the different components self-organize to adapt to the environment and to the task to be performed. Fatigue can be regarded as a determinant affecting how these elements interact, modifying the neuromuscular system’s response during a task. Some studies have analyzed the variability of force production in different contexts (Slifkin and Newell, 2000; Vaillancourt and Newell, 2003; Missenard et al., 2008; Contessa et al., 2009; Singh et al., 2010; Cashaback and Cluff, 2015) identified an increase in variability as relative intensity increased, either due to the effect of age or due to the development of fatigue. In these studies, variability has traditionally been analyzed with measures of dispersion such as standard deviation (Harbourne and Stergiou, 2009) or coefficient of variation (Christou and Carlton, 2001). These measures provide an insight into the magnitude or the amount of variability, and they assume that the variations that occur are random and independent of each other (Caballero et al., 2014).

Nevertheless, these measures of dispersion may not be sensitive to the nature of nested fluctuation patterns of the interdependent elements involved in muscle contraction (Holden, 2005). The multiple time scale of change of the elements implied in motor behavior are often hidden when linear data reduction techniques are applied (Newell et al., 2001). Therefore, as summary statistics would not be neatly applicable to address the complexity of a heterogeneous variable process, measuring the fluctuation changes over time is necessary. The temporal evolution of these fluctuations is known as the structure or dynamics of variability (Caballero et al., 2014). The so-called Non-Linear Tools (NLTs) have been applied to different physiological signals and human movement to analyze the structure of variability (Stergiou, 2004, 2016).

NLTs are mathematical methods that aim to capture variations in how a driving behavior emerges over time. Temporal organization of fluctuations is quantified by the degree to which values emerge in a structured manner across a range of time scales, and its underlying complexity (Harbourne and Stergiou, 2009). Some authors have defined complexity as chaotic temporal variations in a biological system (Yentes et al., 2013) and its structure can be studied through NLTs. Previous studies have linked the loss of complexity to a decrease in the adaptive capacity of the organism, which has led to “the theory of complexity loss” (Goldberger, Peng, et al., 2002), reported in studies on ageing and pathology (Lipsitz and Goldberger, 1992; Slifkin and Newell, 1999; Goldberger, Peng, et al., 2002), and recently extended to studies on fatigue (Beretta-Piccoli et al., 2015). If we understand fatigue as a state in which the organism is in a non-optimal situation we can expect a loss of complexity in the different signals related to force production. Some NLTs are, for example, entropy measures, which estimate the predictability of a signal, i.e., the probability that a data sequence pattern repeats itself in a time series (Pincus, 1991; Richman and Moorman, 2000; Costa et al., 2005). Other NLTs have also been used to study the predictability of a time series, such as the percentage of Determinism (%DET) (Bauer et al., 2017) or the Lyapunov Exponent (LyE), which measures the extent to which the data series represents a similar pattern along time (Wolf et al., 1985).

Additionally, attention must be paid to the tools that analyze the autocorrelation of a time series to understand the complexity of the physiological signals, such as the Detrended Fluctuation Analysis (DFA) (Peng et al., 1995). These tools quantify complexity in different ways, so a decrease in entropy measures [such as approximate entropy (ApEn), sample entropy (SampEn) or fuzzy entropy (FuzzyEn)], in LyE or %DET would reflect a loss of complexity, and an increase in their values would indicate an increase in complexity. Conversely, increasing values of DFA, CrossDet or Cross Shannon Entropy indicate a loss of complexity, and decreasing values indicate an increase in complexity. This NLT has proven to be useful in detecting the flexibility and adaptability of the organism in different scenarios, such as at a physiological level (Goldberger et al., 2002; Goldberger, Peng, et al., 2002; Decker et al., 2011), in postural control (Peng et al., 2009), in injuries detection (Stergiou et al., 2006; Bauer et al., 2017) or learning processes (Barbado et al., 2017). Thus, more complex behaviors have been associated with the individual’s more adaptable and healthy condition. In contrast, more periodic or random behaviors may be associated with a less adaptable or less healthy condition (Stergiou et al., 2006), due to pathology, injury or other factors limiting the organism’s functionality.

Different studies have applied NLTs to analyze fluctuations in force production (Slifkin and Newell, 1999, 2000; Forrest et al., 2014) and electromyography (Farina et al., 2002; González-Izal et al., 2012; Beretta-Piccoli et al., 2015) and kinematic variables (Mann et al., 2015) in fatigue conditions. Given the additional information provided by the NLTs, and that, in some cases, they seem more sensitive than linear measurements (Cavanaugh et al., 2005), applying these methods to force signals for fatigue detection is of interest. In the past few years, there has been an increase in the number of studies that have applied these techniques to force variability concerning fatigue analysis (Missenard et al., 2008; Singh et al., 2010; Cashaback and Cluff, 2015). Therefore, considering the increasing interest in this topic, this review aimed to summarize the findings of studies that analyzed the variability in force production during a muscle fatigue protocol in humans using NLTs. Furthermore, factors such as relative intensity, type of muscle action, recording frequency, and signal processing will also be analyzed to understand their impact on results.

## Methods

### General procedure

This revision was based on the criteria of the PRISMA guidelines (Page et al., 2021). The search, inclusion and exclusion criteria were decided by consensus of all researchers. The search process was carried out in different phases. First, the research question and the search criteria were defined (Supplementary Appendix S1) based on the PICO (participants, interventions, comparisons, outcomes) recommendations (Whiting et al., 2016). Then, the search string was defined, for which different exploratory searches with different keywords were performed. To confirm which combination endorsed the lowest risk of bias, we consulted with experts in systematic reviews, using non-linear tools and in-strength training. Following this, one of the investigators (FGA) performed one first screening to discard titles that were not related to the topic (e.g., articles from the field of engineering). If there was any doubt about a paper, it was added for review at a later stage. In the selection phase by title and abstract, it was necessary to have the consensus of two researchers (FGA and FM) to be included. In the event of a discrepancy a third researcher (RS) was consulted. Once the different articles had been reviewed in depth, it was discussed and decided by consensus of the group whether they could be added to the review.

### Data search and sources

The search was conducted in the following databases: Web of Science (WoS), PubMed, Scopus, and SPORTDiscus (EBSCO). The following search string was used: fatigue AND (entropy OR Lyapunov OR “detrended fluctuation analysis” OR dfa OR “hurst exponent” OR fractal* OR “recurrence quantification” OR autocorrelation). Articles published up to October 2022 were included for this review. The reference manager Mendeley was used to collect and manage the references found, as well as the excel software to manage the results obtained.

### Selection process

The criteria for inclusion in the review were set to answer the target question, which was defined on the basis of the PICO items. Thus, the following inclusion criteria were used: a) published studies with their title and abstract in English, b) original studies that analyzed the effect of fatigue on humans during an action focused on force production, c) studies that applied NLTs on a signal directly related to force production (force signal or kinematic variables).

**FIGURE 1:**
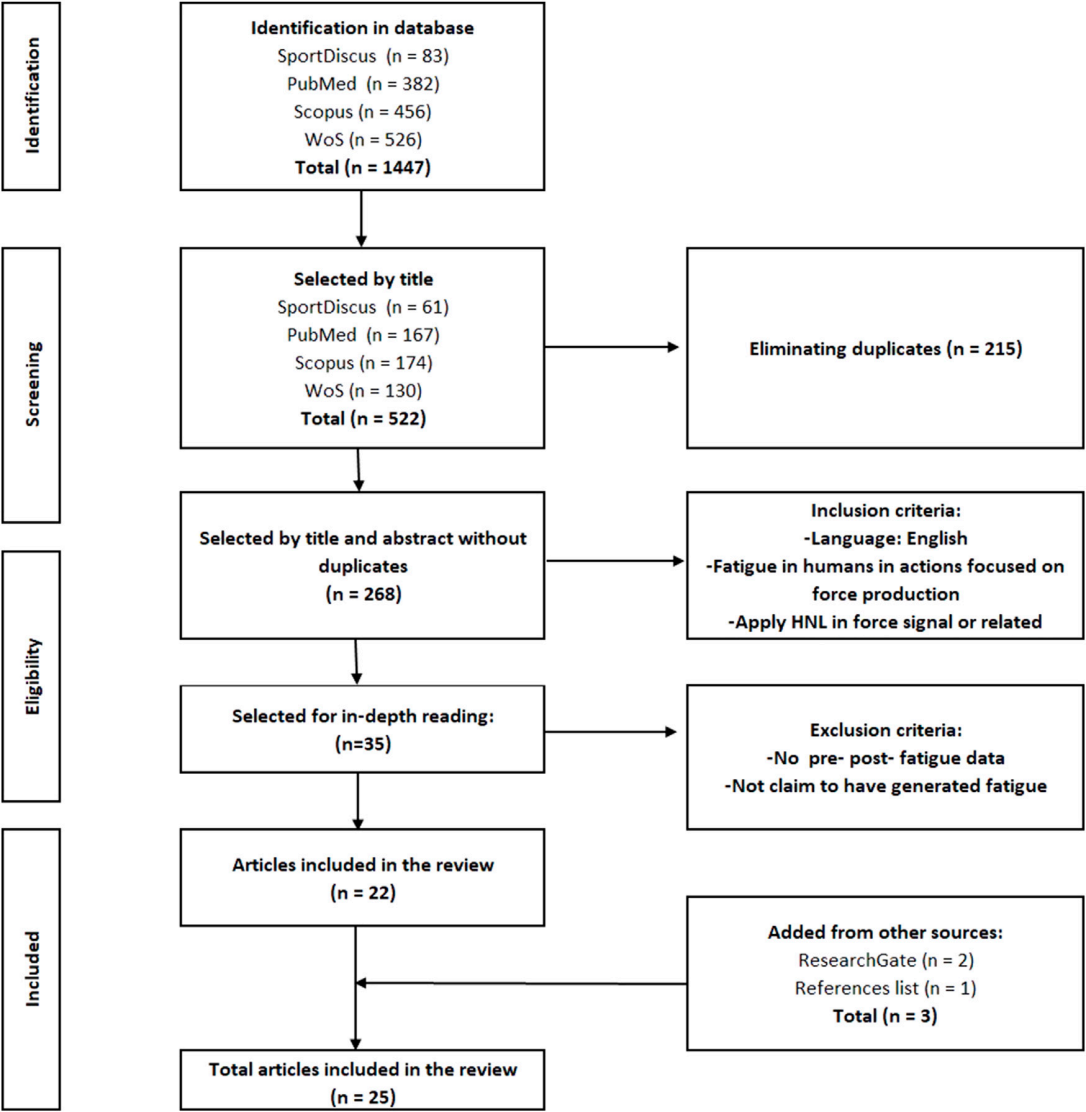
Flowchart of the search strategy.

The findings from each database and the selection process are shown in Figure 1. In addition, the number of articles that were included after reading the title and abstract of each database are also shown in this same figure. Once the duplicates had been eliminated, two reviewers (FGA and FM) agreed on the articles to be included, and when there was any doubt, a third reviewer (RS) was consulted. Additional searches were performed based on the list of references, articles and reviews included, and on the ResearchGate profiles of authors, finding three additional studies that satisfied the inclusion criteria.

### Extraction dates

Data extraction was carried out using a protocol agreed by the authors. The following data were extracted from the selected articles: 1) number of participants; 2) characteristics of the participants; 3) type of fatigue protocol; 3) muscle group targeted by the protocol; 4) intensity and volume of the fatigue protocol; 5) pre- and post-test performed; 6) instruments used for data recording; 7) frequency used for data recording; 8) data processing; 9) NLT used; 10) main results; 11) observations, if relevant.

Data extraction was performed by two reviewers. One performed the main extraction (FGA), and the other confirmed it (CC). The data led to the following sections: 1) main results; 2) physiological explanations for changes in the structure of variability; 3) protocol factors affecting changes in the structure of variability (intensity, volume, type of action, etc.); 4) instrument or data processing factors affecting changes in the structure of variability; 5) other relevant aspects.

### Risk of bias assessment

To analyze the risk of bias of each of the included articles, the Quality Assessment Tool for Before-After (Pre-Post) Studies With No Control Group scale developed by the National Institutes of Health (NIH), was used, which has been recommended for pre-post interventions without a control group (Ma et al., 2020). In addition, ROBIS recommendations (Whiting et al., 2016) were followed to analyze the risk of review bias.

## Results

As a result of the search process, 35 studies were selected from the 268 reviewed and thoroughly read. After analysis according to the above criteria, 22 articles were selected and included in the review. The remaining 12 articles were not included for one of the following reasons: 1) they did not report pre and post fatigue data; 2) they did not claim to have generated fatigue with their protocol. Adding those found from other sources (ResearchGate and references), a total of 25 papers were included in this review.

In terms of risk of bias, most studies had a high NIH score (8 positive, 3 not applicable or not reported, and 1 negative), which is interpreted as high quality. Two studies stand out as potentially slightly biased, namely Bastida-Castillo et al. (2017), as it does not report the data processing completely, and Vázquez et al. (2016), as the sample size seems to be too small. As for the ROSBIS guidelines, the limitations of a narrative review have been found. Supplementary Appendix S2 and S3 reports the results of both assessments.

Twenty-five studies (Gates and Dingwell, 2008; Cowley et al., 2014; Lin et al., 2014; Pethick et al., 2015; Pethick et al., 2016; Vázquez et al., 2016; Bastida-Castillo et al., 2017; Bauer et al., 2017; Pethick et al., 2018a; Pethick et al., 2018b; Cruz-Montecinos et al., 2018; Pethick et al., 2019a; Pethick et al., 2019b; Pethick et al., 2019c; Jiang et al., 2019; Chatain et al., 2020; Guzmán-González et al., 2020; Hollman et al., 2020; Pethick et al., 2020; Tyagi et al., 2020; Zhu et al., 2020; Chatain et al., 2021; Pethick et al., 2021c; Pethick et al., 2021c; Oliveira et al., 2022), which were published between 2008 and 2022, were selected for this review. Table 1 shows the characteristics of the studies, including the sample, the protocol used to cause fatigue, the NLT used, the data acquisition and processing, and the main results.

**TABLE 1:** General characteristics of the studies.

Authors	Sample	Protocol fatigue	NLT	Data acquisition and processing	Main results NLT
Bastida-Castillo et al. (2017)	*n* = 11 trained men	4 × 10 65% RM squat (dynamic)	ApEn	Acceleration data at 1,000 Hz. There is no information about the data processing	Decrement of complexity (decrease of ApEn) together with loss of average propulsive velocity
Bauer et al. (2017)	*n* = 86 (42 men)	Pre-post-test: repeated trunk flexion and extension. Fatigue protocol: isometric trunk extensors to failure	%DET + SampEn	Angular displacement and velocity at 200 Hz. Data were transformed into quaternions and filtered with low-pass Butterworth filter (6 Hz)	Participants without low back pain showed more complex behavior (increase of SampEn and decrease of %DET) in angular velocity after fatigue
	*n* = 59 with low pain (30 men) 39.6 ± 11.6 years				
	*n* = 27 without low pain (12 men) 39.1 ± 12.8 years				
Chatain et al. (2020)	*n* = 11 Healthy active men 24.1 ± 6.6 years	Isometric knee extensor, blocks of 80s at 15% MVC to failure	SampEn	Force sensor data at 2000 Hz were filtered with low-pass Butterworth filter (20 Hz) and down-sampled at 400 Hz. A Dickey-Fuller test and EMD were used	Increase of complexity in the original signal (increase of SampEn), and decrease in complexity (decrease of SampEn) after eliminating non-stationarity
Chatainet al. (2021)	*n* = 38 healthy young adults (19 men) 22.6 ± 2.9 years	Intermittent isometric contractions (8:4s) of the knee extensors at 50% MVC until task failure	RQA (DET)	Force sensor data at 2000 Hz were filtered with low-pass Butterworth filter (20 Hz) and down-sampled at 500 Hz. EMD was used	Reduction of complexity (increase of DET). Men showed more complexity than women
Cowley, Digwell and Gates (2014)	*n* = 20 healthy right-handed adults (11 men) 25 ± 2.2 years	Pre-post-test: Sawing task (dynamic). Fatigue protocol: LIFT at 10%MVC at 0.5 Hz for 3 min or failure. SAW at 25% MVC for 4 min or failure	DFA	Motion analysis system data at 120 Hz was resampled at 1,080 Hz and filtered with low-pass Butterworth filter (6 Hz)	Reduction of complexity (increase of DFA) for error in LIFT and speed in SAW. Men showed more complexity than women. Increment of complexity (decrease of DFA) for speed in LIFT
Cruz-Montecinos, et al. (2018)	*n* = 15 healthy men, age between 18 and 25, right-handed, 21 ± 1 years	Isometric elbow flexor 50% MVC until failure	SampEn	Load cell data at 1000 Hz were filter with low-pass Butterworth filter (12 Hz)	Reduction of complexity (decrease of SampEn)
Gates and Dingwell (2008)	*n* = 14 healthy right-handed (9 men) 27 ± 2.7 years	Sawing task (dynamic) to 15% of this maximum pushing/pulling force until failure	DFA	Motion analysis system data at 60 Hz were resampled at 1,080 Hz and filtered with low-pass Butterworth filter (6 Hz)	Increase of complexity in the original signal (decrease of DFA) in speed and timing error
Guzmán-Gónzalez et al. (2020)	*n* = 12 healthy men right-handed dominance, with sedentary lifestyle 20 ± 2 years	Isometric handgrip flexor 50% MVC until failure	SampEn	Force signal data at 1,500 Hz were filtered with low-pass Butterworth filter (12 Hz)	Reduction of complexity (decrease SampEn)
Hollman, et al. (2020)	*n* = 40 healthy adults (14 men) 23.85 ± 1.7 years	Pre-post-test: 20 step-down task with their preferred stance limb. Fatigue of the hip extensors protocol: isometric trunk extensors to failure. Fatigue sham control group: push-ups until failure	cRQA + cShaEn	Motion analysis system data at 100 Hz were smoothed with a Woltring quintic spline filter (20 mm mean square error)	Reduction of complexity (decrease of cross determinism and mean line) in fatigue hip extensor group
Jiang, et al. (2019)	*n* = 10 healthy men 24 ± 2 years	Isometric contraction of the upper trapezius at 50% MVC until failure	LZC + FEN + LyE	Acceleration data at 1,000 Hz. There is no information about the data processing	Reduction of complexity (decrease of SampEn) in the three conditions (control, self-regulated dual task and regulated dual task)
Lin, Kuo and Hwang (2014)	*n* = 16 healthy men right-handed dominance and between 20 and 24 years	Isometric power gripping in a rhythmic manner at 50–100% MVC until failure	MSE (SampEn)	Digital force gauge data at 1,000 Hz were down-sampled to 100 Hz and filtered with low-pass Butterworth filter (6 Hz)	Increase of complexity (increase of SampEn). In MSE, increase of complexity in high time scale (increase MSE) and reduction of complexity (decrease in low scale time) in low time scale
Oliveira et al. (2022)	*n* = 10 healthy young adults (8 men) 24.9 ± 5.4 years	Pre-post-test: isometric contraction from ankle plantar flexors at 30% MVC for 90s. Fatigue protocol: 5 × 20 unilateral calf raises, 1-min rest between sets	SampEn	Isokinetic dynamometer data at 1,000 Hz. The last 30s were down-sampled to 50 Hz, and analyzed	Reduction of complexity (decrease of SampEn)
Pethick et al. (2020)	*n* = 10 healthy participants (6 men) 25.9 ± 6.7 years	Intermittent isometric (6s: 4s) knee extension at 40% MVC until failure, in ischemic preconditioning and sham treatment groups	ApEn + DFA	Isokinetic dynamometer data at 1,000 Hz. Analyzed the most stable 5s	Reduction of complexity (decrease of ApEn and increase DFA) in both conditions, but with ischemic preconditioning the reduction of complexity was attenuated
Pethick et al. (2015)	*n* = 11 healthy participants (10 men) 25 ± 5.6 years	Intermittent isometric (6s: 4s) knee extension at 40% and maximal MVC until failure	ApEn + SampEn + DFA	Isokinetic dynamometer data at 1,000 Hz. Analyzed the most stable 5s	Reduction of complexity (decrease of ApEn and SampEn and increase DFA) in both conditions, but with submaximal intensity the reduction of complexity was attenuated
Pethick et al. (2016)	*n* = 9 healthy participants (5 men) 25.3 ± 5.8 years	Intermittent isometric (6s: 4s) four trails above CT (approx. 25–35% MVC) and two trials at 50% and 90% of CT	ApEn + DFA	Isokinetic dynamometer data at 1,000 Hz. Analyzed the most stable 5s	Reduction of complexity (decrease of ApEn and increase DFA) in trials above TC but no significant differences below TC
Pethick et al. (2018a)	*n* = 11 healthy participants (7 men) 26.1 ± 6 years	Intermittent isometric (6s: 4s) knee extension at 40% MVC until failure with and without caffeine	ApEn + DFA	Isokinetic dynamometer data at 1,000 Hz. Analyzed the most stable 5s	Reduction of complexity (decrease of ApEn and increase DFA) in both conditions, but with caffeine the reduction of complexity was attenuated
Pethick et al. (2018b)	*n* = 9 healthy participants (5 men) 23.9 ± 5.7 years	Intermittent isometric (6s: 4s) knee extension at 40% MVC until failure with different fatigue conditions ipsilateral, contralateral, ipsilateral with occlusions and contralateral occlusion trials	ApEn + DFA	Isokinetic dynamometer data at 1,000 Hz. Analyzed the most stable 5s	Reduction of complexity (decrease of ApEn and increase DFA) in all conditions, except occlusion ipsi-lateral in which initial complexity was lower
Pethick et al. (2019a)	*n* = 9 healthy participants (7 men) 24.6 ± 5.5 years	Isometric knee extension at 20% and maximal MVC until failure	ApEn + DFA	Isokinetic dynamometer data at 1,000 Hz. Each of the experimental trials was divided into 10 s intervals, using the most stable 5 s	Reduction of complexity (decrease of ApEn and increase DFA) in maximal, but in submaximal only decrease of ApEn
Pethick et al. (2019b)	*n* = 13 healthy participants (10 men) 27.6 ± 6.4 years	Intermittent isometric (6s: 4s) knee extension at 20% and 40% MVC until failure	ApEn + DFA	Isokinetic dynamometer data at 1,000 Hz. Analyzed the most stable 5s	Reduction of complexity (decrease of ApEn and increase DFA) in maximal intensity but non-significant at 20% MV
Pethick et al. (2020)	*n* = 12 healthy participants (7 men) 24.7 ± 4.8 years	Intermittent isometric (6s: 4s) three trails above CT (approx. 25–35% MVC) and four trials at ± 1 and 2 ES of CT	ApEn + DFA	Isokinetic dynamometer data at 1,000 Hz. Analyzed the most stable 5s	Reduction of complexity (decrease of ApEn and increase DFA) at 40% MVC but no significant differences at 20% MVC
Pethick et al. (2021b)	*n* = 11 healthy participants (9 men) 26.3 ± 6 years	Intermittent isometric contractions (6s: 4s) knee extension at 50% MVC, in different knee angles: 30°, 60° and 90° until failure or for 30 min	ApEn + DFA	Isokinetic dynamometer data at 1,000 Hz. Analyzed the most stable 5s	Reduction of complexity (decrease of ApEn and increase DFA) in 90° and 60° angle but no in 30°
Pethick et al. (2019c)	*n* = 10 healthy participants (8 men) 24.8 ± 6.2 years	Intermittent isometric (6s: 4s) knee extension at 50% MVC until failure and eccentric contraction until than MVC >40%	ApEn + DFA	Isokinetic dynamometer data at 1,000 Hz. Analyzed the most stable 5s	Reduction of complexity (decrease of ApEn and increase DFA) in both conditions, but in eccentric recovery it was slower
Tyagi, et al. (2020)	*n* = 42 adults 20 with T1D (9 men) and 22 control (10 men) 22.7 ± 4.5 years	Intermittent isometric (15s: 15s) handgrip at 30% MVC until inability of maintaining contractions or voluntary fatigue	ApEn	Data from the intermediate 10s of the hand-held dynamometer at 1,000 Hz were filtered with a Butterworth low-pass filter (15 Hz)	Reduction of complexity (decrease of ApEn)
Vazquez et al. (2016)	*n* = 7 caucasian men 22.34 ± 3.5 years	Quasi-isometric elbow flexion of 90° at 80% 1RM until failure	DFA	Electro goniometer data at 50 Hz. Amplitude resolution was 0.1° for each extremity do not report to inform about the processing of data	Reduction of complexity (increase DFA)
Zhu et al. (2020)	22 healthy (10 men) and 20 with type 1 diabetes (9 men)	Intermittent isometric (15s: 15s) handgrip at 30% MVC until exhaustion	ApEn	Data from the intermediate 10s of the hand-held dynamometer at 1,000 Hz were filtered with a Butterworth low-pass filter (15 Hz) and of the accelerometer at 45 Hz were filtered with a Butterworth high-pass filter (3 Hz)	Reduction of complexity (decrease of ApEn) in both instrumentals and both groups

Note. RM, repetition maximum; MVC, maximum voluntary contraction; CT, critical torque; SAW, sawing task; LIFT, shoulder flexor; ES, standard error; ApEn, Approximate Entropy; SampEn, Sample Entropy; FEN, fuzzy entropy; MSE, multi scale entropy; cShaEn, cross Shannon Entropy; LZC, Lempel-Ziv complexity; LyE = Lyapunov Exponent; RQA, recurrence quantification analyses; cRQA, cross Recurrence Quantification Analyses; DET, determinism; DFA, detrended fluctuation analysis; EMD, empirical mode decomposition.

### Type of action and intensity

Isometric contractions were carried out in most of these studies (20 out of 25). The participants of 12 studies performed intermittent isometric contractions (Pethick et al., 2015; Pethick et al., 2016; Pethick et al., 2018a; Pethick et al., 2018b; Pethick et al., 2019b; Chatain et al., 2020; Pethick et al., 2020; Tyagi et al., 2020; Zhu et al., 2020; Pethick et al., 2021b; Chatain et al., 2021; Pethick et al., 2021c), in five studies sustained isometric contractions were applied (Cruz-Montecinos et al., 2018; Pethick et al., 2019a; Jiang et al., 2019; Guzmán-González et al., 2020; Oliveira et al., 2022), one study conducted rhythmic isometric contraction (Lin et al., 2014), another study combined intermittent isometric and eccentric contractions (Pethick et al., 2019c), and one study performed a quasi-isometric contraction (Vázquez et al., 2016). The remaining five studies analyzed dynamic contractions (Gates and Dingwell, 2008; Cowley et al., 2014; Bastida-Castillo et al., 2017; Bauer et al., 2017; Hollman et al., 2020) in different movements.

Regarding the volume applied during fatigue protocol, twenty-three studies applied a protocol of fatigue until failure or time limit (Gates and Dingwell, 2008; Cowley et al., 2014; Lin et al., 2014; Pethick et al., 2015; Pethick et al., 2016; Vázquez et al., 2016; Bauer et al., 2017; Pethick et al., 2018a; Pethick et al., 2018b; Cruz-Montecinos et al., 2018; Pethick et al., 2019a; Pethick et al., 2019b; Pethick et al., 2019c; Jiang et al., 2019; Chatain et al., 2020; Guzmán-González et al., 2020; Hollman et al., 2020; Pethick et al., 2020; Tyagi et al., 2020; Zhu et al., 2020; Chatain et al., 2021; Pethick et al., 2021c; Pethick et al., 2021c). In these twenty-three studies, three of them applied a low relative intensity, less than 30% of maximum contraction (Gates and Dingwell, 2008; Cowley et al., 2014; Chatain et al., 2020). Twelve studies applied a sub-maximal relative intensity between 30% and 80% of maximum (Vázquez et al., 2016; Pethick et al., 2018a; Pethick et al., 2018b; Cruz-Montecinos et al., 2018; Pethick et al., 2019c; Jiang et al., 2019; Guzmán-González et al., 2020; Tyagi et al., 2020; Zhu et al., 2020; Pethick et al., 2021b; Chatain et al., 2021; Pethick et al., 2021c). Two different types of relative intensity were analyzed in six studies: low and sub-maximal (Pethick et al., 2016; Pethick et al., 2019b; Pethick et al., 2020), low and maximum (Pethick et al., 2019a) and sub-maximal and maximum (Lin et al., 2014; Pethick et al., 2015). And two studies used body weight (Bauer et al., 2017; Hollman et al., 2020).

The other hand, two studies used different volumes to induce fatigue. Bastida-Castillo, Gómez-Carmona and Pino (2017) conducted a 4 × 10 at 65% of Repetition Maximum (RM). And Oliveira et al. (2022) carry out 5 × 20 at 30% of MVC.

### Type of signal and non-linear tools used

NLTs (see Table 1) were applied in force signal or torque for 17 studies (Lin et al., 2014; Pethick et al., 2015; Pethick et al., 2016; Pethick et al., 2018a; Pethick et al., 2018b; Cruz-Montecinos et al., 2018; Pethick et al., 2019a; Pethick et al., 2019b; Pethick et al., 2019c; Chatain et al., 2020; Guzmán-González et al., 2020; Pethick et al., 2020; Tyagi et al., 2020; Chatain et al., 2021; Pethick et al., 2021c; Pethick et al., 2021c; Oliveira et al., 2022). In one study (Zhu et al., 2020) the signal force was combinate with acceleration force. The other seven studies implemented NLTs in kinematic variables, such as joint angle (Vázquez et al., 2016), distance, speed and timing error (Gates and Dingwell, 2008; Cowley et al., 2014), the coupled hip and knee (Hollman et al., 2020), acceleration signals (Bastida-Castillo et al., 2017; Bauer et al., 2017) and mechanomyography (Jiang et al., 2019). The most common NLT used was entropy measurements, assessed by ApEn (Pethick et al., 2015; Pethick et al., 2016; Bastida-Castillo et al., 2017; Pethick et al., 2018a; Pethick et al., 2018b; Pethick et al., 2019a; Pethick et al., 2019b; Pethick et al., 2019c; Pethick et al., 2020; Tyagi et al., 2020; Zhu et al., 2020; Pethick et al., 2021b; Pethick et al., 2021c), SampEn (Pethick et al., 2015; Bauer et al., 2017; Cruz-Montecinos et al., 2018; Chatain et al., 2020; Guzmán-González et al., 2020; Oliveira et al., 2022), Multi Scale Entropy (MSE) (Lin et al., 2014), Cross Shannon Entropy (Hollman et al., 2020) and FuzzyEn (Jiang et al., 2019). DFA was also applied in 12 studies (Gates and Dingwell, 2008; Cowley et al., 2014; Pethick et al., 2015; Pethick et al., 2016; Vázquez et al., 2016; Pethick et al., 2018a; Pethick et al., 2018b; Pethick et al., 2019a; Pethick et al., 2019b; Pethick et al., 2019c; Pethick et al., 2020; Pethick et al., 2021b; Pethick et al., 2021b). The tools that were used the least were, Lyapunov Exponent (Jiang et al., 2019), Recurrence Quantification Analyses (RQA) (Bauer et al., 2017; Hollman et al., 2020; Chatain et al., 2021), and Lempel-Ziv complexity (Jiang et al., 2019).

### Data acquisition and processing

Most of the studies (19 out of 25) registered signals at 1,000 Hz (Lin et al., 2014; Pethick et al., 2015; Pethick et al., 2016; Bastida-Castillo et al., 2017; Pethick et al., 2018a; Pethick et al., 2018b; Cruz-Montecinos et al., 2018; Pethick et al., 2019a; Pethick et al., 2019b; Pethick et al., 2019c; Jiang et al., 2019; Pethick et al., 2020; Tyagi et al., 2020; Pethick et al., 2021b; Pethick et al., 2021b; Oliveira et al., 2022) or higher (Chatain et al., 2020; Guzmán-González et al., 2020; Chatain et al., 2021). Lower sampling frequencies (lower than 200 Hz) were used in five of the 25 studies. (Gates and Dingwell, 2008; Cowley et al., 2014; Vázquez et al., 2016; Bauer et al., 2017; Hollman et al., 2020). One study combined a high-frequency sample in signal force and low-frequency sample in acceleration signal (Zhu et al., 2020). Regarding data processing, four studies subsampled the time series at 50 Hz (Oliveira et al., 2022), at 100 Hz (Lin et al., 2014), at 400 Hz (Chatain et al., 2020) and at 500 Hz (Chatain et al., 2021) and two oversampled at 1,080 Hz (Gates and Dingwell, 2008; Cowley et al., 2014). As for filters, none were applied in 13 studies. In those studies which did use filters, the most common one was a low-pass filter with different cut-off frequencies: 6 Hz (Gates and Dingwell, 2008; Cowley et al., 2014; Lin et al., 2014; Bauer et al., 2017), 12 Hz (Cruz-Montecinos et al., 2018; Guzmán-González et al., 2020), 15 Hz (Tyagi et al., 2020; Zhu et al., 2020) and 20 Hz (Chatain et al., 2020; Chatain et al., 2021). Only one study (Zhu et al., 2020) applied a high pass filter on accelerations signals, with a cut-off frequency of 3 Hz. Hollman et al. (2020) applied a Woltring quintic spline filter for smooth trajectories. Moreover, Chatain et al. (2020, 2021) used a Dickey-Fuller test for detecting non-stationarity and Empirical Mode Decomposition to obtain a stationary signal.

### Relation between fatigue and force variability

Seventeen studies showed a significant decrease in complexity in all variables in which NLTs were used in the development of fatigue (Pethick et al., 2015; Vázquez et al., 2016; Bastida-Castillo et al., 2017; Pethick et al., 2018a; Pethick et al., 2018b; Cruz-Montecinos et al., 2018; Pethick et al., 2019a; Pethick et al., 2019c; Jiang et al., 2019; Guzmán-González et al., 2020; Hollman et al., 2020; Pethick et al., 2020; Tyagi et al., 2020; Zhu et al., 2020; Chatain et al., 2021; Pethick et al., 2021c; Oliveira et al., 2022). One study (Pethick et al., 2021a) reported a decrease in complexity when the contraction was performed at angles of 60° and 90° at the knee, but not at 30°. Five studies showed different relationships between complexity and fatigue depending on the intensity of the fatigue protocol, the NLTs used, the signal processing or the variable analyzed. Of these five studies two found a decrease in complexity after applying high and sub-maximal relative intensity, but there was no change in complexity with fatigue caused by the application of low intensities (Pethick et al., 2016; Pethick et al., 2019b). Lin, Kuo and Hwang (2014), using MSE, reported a complexity increase at high time scales, and a decrease at low time scales. Chatain et al. (2020) observed a complexity increase due to fatigue in the original signal, but after removing the non-stationarity of the signal, they observed that complexity decreased after fatigue. (Cowley, Dingwell and Gates (2014) found that complexity decreased in the speed variability under general fatigue, but it increased under localized fatigue. On the other hand, two studies showed an increase in complexity due to fatigue (Gates and Dingwell, 2008; Bauer et al., 2017).

## Discussion

This review has examined the current knowledge on the application of NLTs to analyze the relationship between the complexity of force variability and the fatigue state of the organism. Most of the studies reviewed reported a decrease in the complexity of force variability along with the development of fatigue. However, some studies did not report the same results. Therefore, it is necessary to review the proposed mechanisms to explain the possible causes of the decrease in complexity, as well as the factors that apparently modulate the results: intensity, type of contraction, recording frequency, and signal processing.

### Possible mechanisms involved in the loss of complexity

It has been suggested that force variability reflects the interaction between the components of the neuromuscular system (Slifkin and Newell, 1999) and the control loops governing the force output (Fiogbé et al., 2021). Most included studies found a loss of complexity caused by fatigue in tasks with relatively short duration and sub-maximal to maximum relative intensity. In these tasks, fatigue is expected to be related to peripheral factors. Some studies support this claim by shopping one-sidedly between fatigued and non-fatigued limbs (Oliveira et al., 2022; Pethick et al., 2018b). Thus, some studies in this review have interpreted that a loss of complexity may be caused, or at least be affected, by increased metabolic rate (Pethick et al., 2016; Pethick et al., 2018b; Pethick et al., 2019a; Pethick et al., 2021c), reduced force-producing capacity in the motor units (MU) (Pethick et al., 2015; Pethick et al., 2016), or muscle damage caused by eccentric contractions (Pethick et al., 2019c). Although there is no clear explanation Pethick et al. (2021a) speculated that peripheral fatigue mediated by metabolite accumulation may lead to changes in the discharge of motor units, being responsible for changes in complexity.

Secondly, it has also been pointed out that central mechanisms, such as motor unit synchronization and firing rate, affect the loss of complexity. Different studies in this review agree that one of the main mechanisms that may affect this complexity loss is in MU recruitment (Lin et al., 2014; Pethick et al., 2015; Pethick et al., 2018b; Cruz-Montecinos et al., 2018; Pethick et al., 2019a; Pethick et al., 2019b; Pethick et al., 2021a; Pethick et al., 2021a). This is consistent with other studies that have linked changes in organization and activity of MUs to changes in “motor output” fluctuations (Taylor et al., 2003; Adam and De Luca, 2005; Madeleine and Farina, 2008; Skurvydas et al., 2010). Thus, the decrease in the ability to produce force requires a greater synchronization of motor neurons. This increase in synchronization means that the degrees of freedom of the system are reduced, thus reducing complexity. It should be noted that some authors have observed that, although timing is strongly related to variability, it seems that common input also influences changes in variability (Farina and Negro, 2015). On the other hand, some studies have related the loss of complexity to changes in firing rate, which may be modulated by factor such as relative intensity or time contraction. However, literature shows disparate results regarding the relationship between fatigue and the firing rate (Adam and De Luca, 2005; Babault et al., 2006; Boyas and Guével, 2011; Castronovo et al., 2015). Some studies have related this loss of complexity with an increase in the firing rate (Lin et al., 2014; Cruz-Montecinos et al., 2018), while other studies interpret it as a decrease in this rate (Pethick et al., 2015; Bastida-Castillo et al., 2017). These disparate results may be due to the heterogeneity of the protocols used (Boyas and Guével, 2011).

In addition to the above mechanisms, some studies support the influence of central factors on the loss of complexity as they report that with increasing cognitive demands (central level) there was a greater decrease in complexity in force production compared to tasks with lower cognitive demands (Cruz-Montecinos et al., 2018; Guzmán-González et al., 2020; Tyagi et al., 2020). Furthermore, caffeine consumption, which affects the central nervous system, has also been shown to slow down the loss of complexity that is caused by the onset of fatigue (Pethick et al., 2018a).

Different authors have discussed the relationship between fatigue of central and peripheral origin, with one affecting the other (Gandevia, 2001; Boyas and Guével, 2011). It has been suggested that changes at the peripheral level produced by fatigue are compensated by changes at the central level (Taylor and Gandevia, 2008). For example, when the capacity to produce force is reduced due to alterations at the peripheral level, the increase in active MU is activated as a compensation mechanism (Gandevia, 2001; Pethick et al., 2015; Pethick et al., 2019b). It has also been suggested that afferent outputs from the muscle affect central levels (Taylor and Gandevia, 2008; Boyas and Guével, 2011), so changes in a central level due to fatigue would be useful to protect the muscle from further peripheral fatigue (Gandevia, 2001). Therefore, attempting to understand changes in force complexity solely based on of the central or peripheral origin of fatigue may not adequately portray the process that triggers fatigue mechanisms, which are not independent elements, but are interconnected. Furthermore, Lin et al. (2014) considered that the loss of complexity observed at the shorter time scale (1–5) could be related to the increase in active motoneurons d, and to the increase in excitatory impulses coming from the central nervous system compensating for the loss of force. Meanwhile, the increase in complexity observed over longer time scales (25–40) could result from motor noises associated with fatigue. The opposing time-scale trends in completeness with the development of fatigue could reflect the interaction of at least two regulatory systems, one voluntary and one involuntary, operating over a wide range of time scales, and it could reflect the central and peripheral mechanisms mentioned above. Thus, as suggested by Pethick et al., (2021b), both peripheral and central processes appear to be involved in the loss of complexity at the onset of fatigue. We suggest understanding this as a feedback loop in which different peripheral triggers leading to a decrease in force production are compensated by central mechanisms, resulting in a decrease in complexity. This could be caused by the reduction of the degrees of freedom needed to meet the task demands. Thus, the complexity in the different force production signals would decrease due to fatigue, indicating that the organism is in a situation where it is less flexible and has a reduced adaptive capacity.

### Intensity and type of contraction

The intensity of the contraction seems to be one relevant factor involved in the change in complexity due to fatigue. Most studies assessing actions at sub-maximal or maximum intensity have reported a decrease in complexity after the fatigue protocol (Pethick et al., 2015; Pethick et al., 2016; Vázquez et al., 2016; Bastida-Castillo et al., 2017; Pethick et al., 2018a; Pethick et al., 2018b; Cruz-Montecinos et al., 2018; Pethick et al., 2019a; Pethick et al., 2019b; Pethick et al., 2019c; Guzmán-González et al., 2020; Pethick et al., 2020; Tyagi et al., 2020; Zhu et al., 2020; Chatain et al., 2021; Pethick et al., 2021c; Oliveira et al., 2022). One study (Pethick et al., 2021a) reported a loss of complexity at submaximal intensity (50% MVC) in knee extension when the knee was at 90° and 60° of extension (0° full extension), but not at 30°. The authors state that in other studies, to achieve similar responses at 90° of extension, the relative intensity had to be increased to 30°.

Only one study showed contradictory results according to the time scale analyzed using MSE (Lin et al., 2014). It should be noted that this type of analysis allows us to analyze how complexity behaves at different temporal scales, which could be related to different system elements involved. Moreover, according to Stergiou (2016), if entropy (SampEn in this case) tends to decrease with increasing time scale, it could mean that the relevant information is only at low time scales, which could be related to the voluntary control mentioned above. In this case, the contradictory results may be because they performed sinusoidal isometric contractions, as reported by Vaillancourt and Newell (2003) who found different trends depending on whether constant or sinusoidal isometric actions were performed. It may also be the case that, while entropy measures typically analyze a single time scale, the MSE examines different scales within the same time series (Costa et al., 2005). As mentioned above, this could be related to the type of control (voluntary or involuntary). Thus, it may be that this type of actions may lead to an increase in complexity in involuntary control, but a decrease in complexity in voluntary control.

Papers that analyzed exercises carried out at low relative intensity did not follow the aforementioned trend between fatigue and complexity, revealing no significant differences (Pethick et al., 2016; Pethick et al., 2019a; Pethick et al., 2019b) or even showing an increase in complexity with the development of fatigue (Gates and Dingwell, 2008). These differences depending on relative intensity could be because the fact that the organism would not be in a sufficiently compromised situation to show a loss of complexity during low-intensity exercises. For instance, it has been found that there is an inverse relationship between metabolic rate and complexity (Pethick et al., 2019b). If the metabolic rate is not increased enough at low relative intensity, complexity may not decrease. The same applies to muscle oxygenation (Pethick et al., 2019b) and changes in movement patterns depending on the relative intensity required (Gates and Dingwell, 2008; Cowley et al., 2014). Consequently, if these changes are irrelevant, the central control mechanisms will adjust the system’s requirements without significant loss of complexity. Pethick et al. (2016) noticed that loss of complexity happens because of intensities exceeding the critical torque, which is approximately 20–25% of the MVC. In another study, it was found that this critical torque is not an exact threshold but a transition phase (Pethick et al., 2021b). It is important to keep this in mind, since below this critical torque or critical power, the organism may not have important changes in homeostasis (Enoka and Duchateau, 2016). Moreover, as noted above, they may even be sensitive to the angle at which the force is produced, as the relative intensity for an effort will change as a function of factors such as the length of the cross-bridge or lever arm, for example. Thus, while they may be a development of fatigue, it may not be significant enough to affect complexity or the effect may be minimal. This could explain why the NLTs seem to be less sensitive at low intensities, thus showing no loss of complexity.

Another aspect that seems to affect the results is the type of muscle activation. Most studies measured isometric actions at sub-maximal and high intensities, resulting in a decrease in complexity due to fatigue (Pethick et al., 2015; Pethick et al., 2016; Vázquez et al., 2016; Pethick et al., 2018a; Pethick et al., 2018b; Cruz-Montecinos et al., 2018; Pethick et al., 2019a; Pethick et al., 2019b; Jiang et al., 2019; Chatain et al., 2020; Guzmán-González et al., 2020; Pethick et al., 2020; Tyagi et al., 2020; Zhu et al., 2020; Pethick et al., 2021a; Chatain et al., 2021; Pethick et al., 2021c; Oliveira et al., 2022). But only one study, Pethick et al. (2019c), analyzed eccentric and isometric contractions, reporting greater fatigue and muscle damage after the eccentric contractions, and longer recovery time in eccentric actions (Pethick et al., 2019c). In that study, linear measures of variability, such as standard deviation and coefficient of variation, returned to baseline in a shorter period of time (10 and 30 min, respectively) than measures of complexity. ApEn and DFA maintained low complexity values for a longer period showing that the organism had not yet recovered, although it is possible that it is due to a greater extent to muscle damage. Thus, this study also suggests that the NLTs are more sensitive than traditional measures of variability.

In addition, there were five studies that analyzed dynamic actions and only two of these five studies found a loss of complexity due to fatigue (Bastida-Castillo et al., 2017; Hollman et al., 2020). The rest of them showed controversial results. Two studies found an increase in complexity (Gates and Dingwell, 2008; Bauer et al., 2017), one study found an increase or decrease of complexity depending on the variable analyzed (Cowley et al., 2014). It has to be pointed out that two of the studies that found an increase of complexity due to fatigue, conducted actions at low intensities (Gates and Dingwell, 2008; Cowley et al., 2014), and it is possible that these results are mediated by relative intensity, as mentioned above. Is should also be noted that two of the studies (Bauer et al., 2017; Hollman et al., 2020) used body weight loading as part of the fatigue protocol and found opposite results. These studies found contrary results, with Bauer et al. (2017) reporting an increase in complexity, while Hollman et al. (2020) reported a decrease. Both fatigued the lumbar musculature, using the Biering-Sorensen test, in the fatiguing protocol, but in the pre- and post-tests there was a greater involvement of the hip extensors. It should be noted that Hollman et al. (2020) compared the lumbar muscle fatigue protocol with a control protocol (push-up to failure protocol) and reported that changes in complexity only occurred in the lumbar muscle protocol, which was more specific than the other. We interpret that these controversial results in dynamic actions have three causes. Firstly, the methodological heterogeneity of the studies. Secondly, because of the influence of relative intensity, as mentioned above. Finally, due to the differences between dynamic and isometric actions. Differences in the involvement of the central and peripheral levels in fatigue have been reported, depending on whether the actions are isometric or concentric (Allen et al., 2008). Furthermore, given the nature of dynamic actions, it is to be expected that these have a higher non-stationarity component than isometric actions. This increased non-stationarity will affect the results, since some NLTs follow algorithms that assume a higher degree of stationarity (Stergiou, 2016). Thus, in order to obtain robust and reliable results, some of the HNLs mentioned above need stationarity in the time series in which they are applied (e.g., the LyE and entropy measures) (Caballero et al., 2014). Therefore, signal processing can play an important role in the study of complexity and its relation to fatigue.

### Influence of signal recording and processing

Both the nature of the signal and its processing (e.g., sample frequency, filtering) are significant factors when using NLTs (Stergiou, 2016). In order to select the most appropriate sample frequency, both the purpose of the analysis and the system’s behavior (Stergiou, 2004, 2016) should be considered. Although the studies analyzed in this review have not looked into this matter, the results seem to suggest that it is advisable to use high recording frequencies to capture the dynamics of fluctuations in force production. Forrest et al. (2014) found that frequencies below 200 Hz were not suitable for ApEn analysis on force signals. It appears that recording frequencies below this threshold may modify the shape of the recorded signal and prevent capturing the dynamics of variations in force production. Most of the studies in this review that used recording frequencies higher than 200 Hz (n = 20) reported a loss of complexity along with the development of fatigue (Pethick et al., 2015; Pethick et al., 2016; Pethick et al., 2018a; Pethick et al., 2018b; Cruz-Montecinos et al., 2018; Pethick et al., 2019a; Pethick et al., 2019b; Pethick et al., 2019c; Guzmán-González et al., 2020; Pethick et al., 2020; Zhu et al., 2020; Pethick et al., 2021b; Pethick et al., 2021c), exception the study by Chatain et al. (2020). Although Chatain et al. (2020) found an increase in complexity in the original signal after the fatigue protocol, they found a decrease in complexity when the non-stationarity of the signal was eliminated. Studies using recording frequencies of 200 Hz or lower (*n* = 6) reported results in different directions (Lin et al., 2014; Vázquez et al., 2016; Bauer et al., 2017; Hollman et al., 2020; Zhu et al., 2020; Oliveira et al., 2022). Two of these studies oversampled the signal, increasing the length of the data sets obtained in the record, and reported that fatigue caused an increase in complexity in some measured variables (Gates and Dingwell, 2008; Cowley et al., 2014), and a decrease in others (Cowley et al., 2014). Furthermore, it has been observed that in previous reviews on the use of NLTs, these have shown the disadvantage of obtaining non-reliable results when the length of time series is artificially increased as this signal processing may significantly influence the results of the analysis, which is not advisable (Stergiou, 2016).

Once the recording frequency has been determined, the application of a filter can be considered. To consider the need for a filter we have to know the characteristics of the signal (e.g., how noisy it is). On this basis the type of filter will also be decided. While in some cases it has been recommended to avoid filtering signals to analyze the structure of the variability of a time series (Caballero et al., 2014; Stergiou, 2016), one of the most commonly applied treatments is the low-pass filter to prevent the occurrence of “unwanted” noise. The analyzed studies reported low-pass filters with cut-off frequencies of 6 Hz (Gates and Dingwell, 2008; Cowley et al., 2014; Lin et al., 2014; Bauer et al., 2017), 12 Hz (Cruz-Montecinos et al., 2018; Guzmán-González et al., 2020), 15 Hz (Tyagi et al., 2020; Zhu et al., 2020), 20 Hz (Chatain et al., 2020; Chatain et al., 2021), and in one of the studies a Woltring quintic spline filter (Hollman et al., 2020) was used. Two of these studies showed a clear increase in complexity (Gates and Dingwell, 2008; Bauer et al., 2017), three showed mixed results (Cowley et al., 2014; Lin et al., 2014; Chatain et al., 2020), and five revealed a decrease in complexity (Cruz-Montecinos et al., 2018; Guzmán-González et al., 2020; Hollman et al., 2020; Tyagi et al., 2020; Chatain et al., 2021). It seems that when the filter cut-off is made at a frequency of 6 Hz the results become less consistent. This could be because, as some authors have pointed out (Singh et al., 2010; Novak and Newell, 2017), frequencies ≤4 Hz reflect voluntary control loops, while frequencies between 8 and 12 Hz reflect involuntary control loops such as physiological tremor. Based on this, Zhu et al. (2020) used a high-pass filter with a 3 Hz cutoff frequency to analyze the complexity of the tremor, reporting loss of complexity in both the force signal and tremor in the acceleration signal. Thus, if we use low-pass filters above 12 Hz it is possible that the signal hardly varies at all, and therefore the results are more consistent. On the other hand, it can be interesting to analyze the signals at different frequency widths to find out how the different voluntary and involuntary control systems affect the complexity of the signal.

Finally, it should be noted that NLTs are sensitive to the stationarity of the time series (Peng et al., 2009; Caballero et al., 2013), which may have influenced the results reported by the studies reviewed. Two studies (Chatain et al., 2020; Chatain et al., 2021) applied Empirical Mode Decomposition (EMD) to reduce the non-stationarity of the signal. The comparison between the original and the treated signal was only performed in one of them (Chatain et al., 2020). In that study the authors observed that complexity decreased due to fatigue when non-stationarity was reduced, while it increased in the original signal. This was observed at low intensities (15% MVC), and it suggests that the non-stationarity of the signal affects the sensitivity of NLTs, although further research is needed to explore this methodological aspect in the application of these tools when analyzing force variability. Moreover, non-stationarity can be expected to have a bigger impact on dynamic actions. Thus, it would be interesting to verify if applying this type of method improves the robustness and reliability of NLTs to analyze fatigue in non-isometric contractions.

## Limitations, conclusion and future perspectives

The main limitations of this review were the heterogeneity of protocols, both of the NLTs used and of the different signal treatments, which makes it difficult to draw solid conclusions. The low number of studies on dynamic actions was another significant limitation, which indicating the difficulty of performing non-linear analyses on this type of actions. In addition to the above limitations, some factors can modify the complexity values, such as pathologies (Bauer et al., 2017; Tyagi et al., 2020), or intrinsic characteristics of the participant such as gender (Duan et al., 2018; Chatain et al., 2021). Therefore, these variables should be considered in future work relating fatigue to complexity.

**FIGURE 2:**
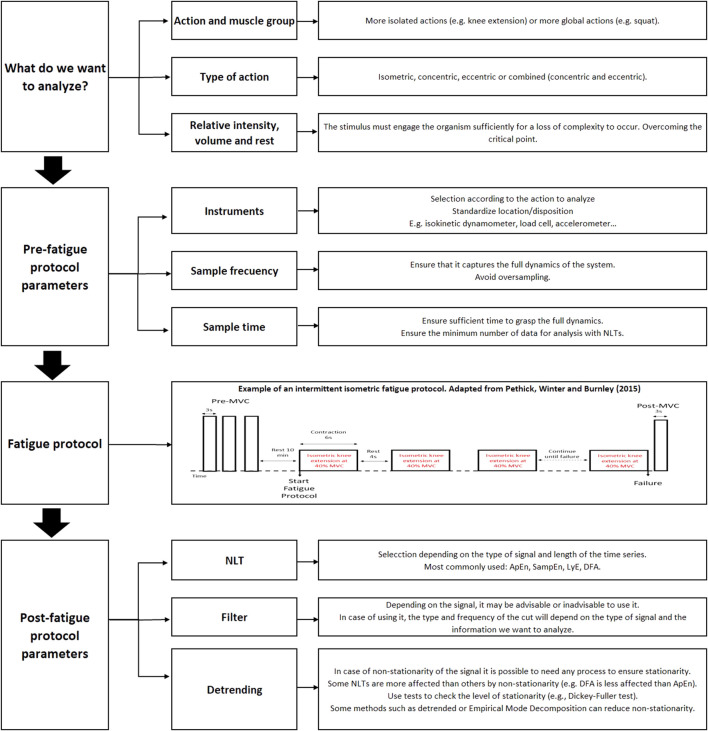
Flowchart with the steps to analyse the structure of variability in kinetic and kinematic signals.

As mentioned above, a relationship has been suggested between fatigue and loss of complexity in isometric actions at a relative intensity that engages the body (above the critical point). This loss of complexity appears to reflect changes at the central level that occur to compensate for alterations at the peripheral level. This clear relationship has not been observed in dynamic actions, where factors such as non-stationarity may hinder the application of NLTs. This review has highlighted the importance of proper selection of the recording method and signal processing. Thus, the following are suggested as practical recommendations for analyzing force variability (see Figure 2). Based on the studies reviewed, it is suggested that recording frequencies of 200 Hz are adequate to capture the dynamics of the system. Low-pass filters with cut-off frequencies above 12 Hz do not seem to be particularly influential on the results. And it is also possible that analyzing using filters with cut-off ranges with frequencies of 12 Hz allows different aspects of force control to be studied (e.g., <4 Hz to study voluntary control or 8–12 Hz to study involuntary control). However, non-stationarity should be a factor to be considered, especially in rhythmic isometric or dynamic actions. Methods such as the EMD can be effective in eliminating the non-stationarity of the signal. In addition, it may be advisable to use methods that allow to analyze the structure on different time scales (e.g., MSE), as the way these methods develop in different structures of the system can then be analyzed. Finally, as recommended by some authors (Harbourne and Stergiou, 2009; Caballero et al., 2014; Stergiou, 2016), the use of different NLTs is also advisable, since these can measure complementary aspects of variability (regularity, autocorrelation, etc.).

Further studies are needed in two directions. On the one hand, an in-depth study of dynamic contractions, as they are the most frequently performed in sport and in everyday life, and are the ones about which we have the least information. In this way, it will be possible to know if there is a relationship between fatigue and the loss of complexity in these contractions, as well as to know the correct methodology to apply. On the other hand, it would be convenient to study whether it is possible to use these tools in dynamic and isometric contractions to monitor fatigue in training sessions. In this way, it would be possible to control the state of fatigue in which the organism finds itself, allowing safer and more efficient training programs to be carried out. In addition, since no study specifically addresses the neural mechanisms causing the loss of complexity, it would be desirable to add measures to understand the neural contributions to the loss of complexity, e.g., transcranial magnetic stimulation. It would be interesting to conduct studies with the aim of analyzing the underlying mechanisms. To this end, measures can be added to understand the neural contributions to the loss of complexity, e.g., transcranial magnetic stimulation.

## Data Availability

The original contributions presented in the study are included in the article/Supplementary Material, further inquiries can be directed to the corresponding author.

## References

[B1] Adam A. De Luca C. J. (2005). Firing rates of motor units in human vastus lateralis muscle during fatiguing isometric contractions. J. Appl. Physiol. 99 (1), 268–280. 10.1152/japplphysiol.01344.2004 16036904

[B2] Allen D. G. Lamb G. D. Westerblad H. (2008). Skeletal muscle fatigue: Cellular mechanisms. Physiol. Rev. 88 (1), 287–332. 10.1152/physrev.00015.2007 18195089

[B3] Almonroeder T. G. Tighe S. M. Miller T. M. Lanning C. R. (2020). The influence of fatigue on decision-making in athletes: A systematic review. Sports Biomech. 19 (1), 76–89. 10.1080/14763141.2018.1472798 29902127

[B4] Ament W. Verkerke G. (2009). Exercise and fatigue. Sports Med. 39 (5), 389–422. 10.2165/00007256-200939050-00005 19402743

[B5] Babault N. Desbrosses K. Fabre M. S. Michaut A. Pousson M. (2006). Neuromuscular fatigue development during maximal concentric and isometric knee extensions. J. Appl. Physiol. 100 (3), 780–785. 10.1152/japplphysiol.00737.2005 16282433

[B6] Badillo J. J. G. J. R. S. (2002). Bases de la programación del entrenamiento de fuerza. Barcerlona: INDE.

[B7] Barbado D. Caballero Sanchez C. Moreside J. Vera-Garcia F. J. Moreno F. J. (2017). Can the structure of motor variability predict learning rate? J. Exp. Psychol. Hum. Percept. Perform. 43 (3), 596–607. 10.1037/xhp0000303 28095006

[B8] Bastida-Castillo A. Gómez-Carmona C. D. Pino J. (2017). Relationship Between Aproximate Entropy and Mean Propulsive Velocity Loss During Half Squat Exercise desde la etapa formativa. View project Inertial Device Assessment for Load Quantification in Sport View project. Rev. Kronos 16 (2).

[B9] Bauer C. M. Rast F. M. Ernst M. J. Meichtry A. Kool J. Rissanen S. M. (2017). The effect of muscle fatigue and low back pain on lumbar movement variability and complexity. J. Electromyogr. Kinesiol. 33, 94–102. 10.1016/j.jelekin.2017.02.003 28226298

[B10] Beretta-Piccoli M. D'Antona G. Barbero M. Fisher B. Dieli-Conwright C. M. Clijsen R. (2015). Evaluation of central and peripheral fatigue in the quadriceps using fractal dimension and conduction velocity in young females. PLoS ONE 10 (4), 01239211–e124015. 10.1371/journal.pone.0123921 PMC4400165 25880369

[B11] Boyas S. Guével A. Guevel A. (2011). Neuromuscular fatigue in healthy muscle: Underlying factors and adaptation mechanisms. Ann. Phys. Rehabil. Med. 54 (2), 88–108. 10.1016/j.rehab.2011.01.001 21376692

[B12] Caballero C. Barbado D. Moreno F. J. (2014). Non-linear tools and methodological concerns measuring human movement variability: An overview. Eur. J. Hum. Mov. 32, 61–81.

[B13] Caballero C. Barvado D. Moreno F. J. (2013). El procesado del desplazamiento del centro de presiones para el estudio de la relación complejidad/rendimiento observada en el control postural en bipedestación. Med. del deporte 6 (3), 101–107.

[B14] Carroll T. J. Taylor J. L. Gandevia S. C. (2017). Recovery of central and peripheral neuromuscular fatigue after exercise. J. Appl. Physiol. 122 (5), 1068–1076. 10.1152/japplphysiol.00775.2016 27932676

[B15] Cashaback J. G. A. Cluff T. (2015). Increase in joint stability at the expense of energy efficiency correlates with force variability during a fatiguing task. J. Biomech. 48 (4), 621–626. 10.1016/j.jbiomech.2014.12.053 25597814

[B16] Castronovo A. M. Negro F. Conforto S. Farina D. (2015). The proportion of common synaptic input to motor neurons increases with an increase in net excitatory input. J. Appl. Physiol. 119 (11), 1337–1346. 10.1152/japplphysiol.00255.2015 26404614

[B17] Cavanaugh J. T. Guskiewicz K. M. Stergiou N. (2005). A nonlinear dynamic approach for evaluating postural control: New directions for the management of sport-related cerebral concussion. Sports Med. 35 (11), 935–950. 10.2165/00007256-200535110-00002 16271008

[B18] Chatain C. Gruet M. Vallier J. M. Ramdani S. (2020). Effects of nonstationarity on muscle force signals regularity during a fatiguing motor task. IEEE Trans. Neural Syst. Rehabil. Eng. 28 (1), 228–237. 10.1109/TNSRE.2019.2955808 31765316

[B19] Chatain C. Ramdani S. Vallier J. M. Gruet M. (2021). Recurrence quantification analysis of force signals to assess neuromuscular fatigue in men and women. Biomed. Signal Process. Control 68, 102593. 10.1016/j.bspc.2021.102593

[B20] Christou E. Carlton L. G. (2001). Old adults exhibit greater motor output variability than young adults only during rapid discrete isometric contractions. J. Gerontol. A Biol. Sci. Med. Sci. 56 (12), 524–532. 10.1093/gerona/56.12.b524 11723145

[B21] Contessa P. Adam A. De Luca C. J. (2009). Motor unit control and force fluctuation during fatigue. J. Appl. Physiol. 107 (1), 235–243. 10.1152/japplphysiol.00035.2009 19390005 PMC2711782

[B22] Cortes N. Onate J. Morrison S. (2014). Differential effects of fatigue on movement variability. Gait Posture 39 (3), 888–893. 10.1016/j.gaitpost.2013.11.020 24370441 PMC3960345

[B23] Costa M. Goldberger A. L. Peng C.-K. (2005). Multiscale entropy analysis of complex physiologic time series. Phys. Rev. Lett. 89 (6), 068102–068108. 10.1103/PhysRevLett.89.068102 12190613

[B24] Cowley J. C. Dingwell J. B. Gates D. H. (2014). Effects of local and widespread muscle fatigue on movement timing. Exp. Brain Res. 232 (12), 3939–3948. 10.1007/s00221-014-4020-z 25183157 PMC4241184

[B25] Cruz-Montecinos C. Calatayud J. Iturriaga C. Bustos C. Mena B. Espana-Romero V. (2018). Influence of a self-regulated cognitive dual task on time to failure and complexity of submaximal isometric force control. Eur. J. Appl. Physiol. 118 (9), 2021–2027. 10.1007/s00421-018-3936-6 29987354

[B26] Decker L. M. Moraiti C. Stergiou N. Georgoulis A. D. (2011). New insights into anterior cruciate ligament deficiency and reconstruction through the assessment of knee kinematic variability in terms of nonlinear dynamics. Knee Surg. Sports Traumatol. Arthrosc. 19 (10), 1620–1633. 10.1007/s00167-011-1484-2 21445594

[B27] Duan X. Rhee J. Mehta R. K. Srinivasan D. (2018). Neuromuscular control and performance differences associated with gender and obesity in fatiguing tasks performed by older adults. Front. Physiol. 9, 800–814. 10.3389/fphys.2018.00800 30018563 PMC6037858

[B28] Enoka R. M. Duchateau J. (2008). Muscle fatigue: What, why and how it influences muscle function. J. Physiol. 586 (1), 11–23. 10.1113/jphysiol.2007.139477 17702815 PMC2375565

[B29] Enoka R. M. Duchateau J. (2016). Translating fatigue to human performance. Med. Sci. Sports Exerc. 48 (11), 2228–2238. 10.1249/MSS.0000000000000929 27015386 PMC5035715

[B30] Farina D. Fattorini L. Felici F. Filligoi G. (2002). Nonlinear surface EMG analysis to detect changes of motor unit conduction velocity and synchronization. J. Appl. Physiol. 93 (5), 1753–1763. 10.1152/japplphysiol.00314.2002 12381763

[B31] Farina D. Negro F. (2015). Common synaptic input to motor neurons, motor unit synchronization, and force control. Exerc. Sport Sci. Rev. 43 (1), 23–33. 10.1249/JES.0000000000000032 25390298

[B87] Fiogbé E. Vassimon-Barroso V. Catai A. M. de Melo R. C. Quitério R. J. Porta A. (2021). Complexity of knee extensor torque: Effect of aging and contraction intensity. The Journal of Strength & Conditioning Research 35 (4), 1050–1057. 30289867 10.1519/JSC.0000000000002888

[B32] Forrest S. M. Challis J. H. Winter S. L. (2014). The effect of signal acquisition and processing choices on ApEn values: Towards a “gold standard” for distinguishing effort levels from isometric force records. Med. Eng. Phys. 36 (6), 676–683. 10.1016/j.medengphy.2014.02.017 24725708

[B33] Gandevia S. C. (2001). Spinal and supraspinal factors in human muscle fatigue. Physiol. Rev. 81 (4), 1725–1789. 10.1152/physrev.2001.81.4.1725 11581501

[B34] Gates D. H. Dingwell J. B. (2008). The effects of neuromuscular fatigue on task performance during repetitive goal-directed movements. Exp. Brain Res. 187 (4), 573–585. 10.1007/s00221-008-1326-8 18327575 PMC2825378

[B35] Goldberger A. L. Amaral L. A. N. Hausdorff J. M. Ivanov P. C. Peng C. K. Stanley H. E. (2002). Fractal dynamics in physiology: Alterations with disease and aging. Proc. Natl. Acad. Sci. U. S. A. 99 (1), 2466–2472. 10.1073/pnas.012579499 11875196 PMC128562

[B36] Goldberger A. L. Peng C. K. Lipsitz L. A. (2002). What is physiologic complexity and how does it change with aging and disease? Neurobiol. Aging 23 (1), 23–26. 10.1016/S0197-4580(01)00266-4 11755014

[B37] González-Izal M. Malanda A. Gorostiaga E. Izquierdo M. (2012). Electromyographic models to assess muscle fatigue. J. Electromyogr. Kinesiol. 22 (4), 501–512. 10.1016/j.jelekin.2012.02.019 22440555

[B38] Guzmán-González B. Bustos-Briones C. Calatayud J. Tapia C. Torres-Elgueta J. Garcia-Masso X. (2020). Effects of dual-task demands on the complexity and task performance of submaximal isometric handgrip force control. Eur. J. Appl. Physiol. 120 (6), 1251–1261. 10.1007/s00421-020-04357-x 32242254

[B39] Harbourne R. T. Stergiou N. (2009). Movement variability and the use of nonlinear tools: Principles to guide physical therapist practice. Phys. Ther. 89 (3), 267–282. 10.2522/ptj.20080130 19168711 PMC2652347

[B40] Holden J. G. (2005). “‘Chapter 6: Gauging the fractal dimension of response times from cognitive tasks’,” in Contemporary nonlinear methods for behavioral scientists: A webbook tutorial, 267–318.

[B41] Hollman J. H. Beise N. J. Fischer M. L. Stecklein T. L. (2020). Hip extensor fatigue alters hip and knee coupling dynamics during single-limb step-downs: A randomized controlled trial. J. Biomech. 100, 109583. 10.1016/j.jbiomech.2019.109583 31870658

[B42] Jiang W. Xia C. Zhang Y. Xie J. Feng W. (2019). “Research on muscle fatigue trend via nonlinear dynamic feature analysis of mechanomyography signal,” in 2019 IEEE 4th International Conference on Signal and Image Processing ICSIP, Wuxi, China, 19-21 July 2019, 669–673. 10.1109/SIPROCESS.2019.8868859

[B43] Lin Y. T. Kuo C. H. Hwang I. S. (2014). Fatigue effect on low-frequency force fluctuations and muscular oscillations during rhythmic isometric contraction. PLoS ONE 9 (1), e85578. 10.1371/journal.pone.0085578 24465605 PMC3897466

[B44] Lipsitz L. A. Goldberger A. L. (1992). Loss of “complexity” and aging: Potential applications of fractals and chaos theory to senescence. JAMA J. Am. Med. Assoc. 267 (13), 1806–1809. 10.1001/jama.1992.03480130122036 1482430

[B45] Ma L. L. Wang Y. Y. Yang Z. H. Huang D. Weng H. Zeng X. T. (2020). Methodological quality (risk of bias) assessment tools for primary and secondary medical studies: What are they and which is better? Mil. Med. Res. 7 (1), 7–11. 10.1186/s40779-020-00238-8 32111253 PMC7049186

[B46] Madeleine P. Farina D. (2008). Time to task failure in shoulder elevation is associated to increase in amplitude and to spatial heterogeneity of upper trapezius mechanomyographic signals. Eur. J. Appl. Physiol. 102 (3), 325–333. 10.1007/s00421-007-0589-2 17943307

[B47] Mann R. Malisoux L. Urhausen A. Statham A. Meijer K. Theisen D. (2015). The effect of shoe type and fatigue on strike index and spatiotemporal parameters of running. Gait Posture 42 (1), 91–95. 10.1016/j.gaitpost.2015.04.013 25953506

[B48] Missenard O. Mottet D. Perrey S. (2008). Muscular fatigue increases signal-dependent noise during isometric force production. Neurosci. Lett. 437 (2), 154–157. 10.1016/j.neulet.2008.03.090 18440146

[B49] Newell K. M. Liu Y. T. Mayer-Kress G. (2001). Time scales in motor learning and development. Psychol. Rev. 108 (1), 57–82. 10.1037/0033-295X.108.1.57 11212633

[B50] Novak T. Newell K. M. (2017). Physiological tremor (8–12 Hz component) in isometric force control. Neurosci. Lett. 641, 87–93. 10.1016/j.neulet.2017.01.034 28109777

[B51] Oliveira J. Casanova N. Gomes J. S. Pezarat-Correia P. Freitas S. Vaz J. R. (2022). Changes in torque complexity and maximal torque after a fatiguing exercise protocol. Sports Biomech. 2022, 1–13. 10.1080/14763141.2022.2067588 35485846

[B52] Page M. J. Grimshaw J. M. Hrobjartsson A. Lalu M. M. Li T. Loder E. W. (2021). The prisma 2020 statement: An updated guideline for reporting systematic reviews. Med. Flum. 57 (4), 444–465. 10.21860/medflum2021_264903

[B53] Peng C. K. Costa M. Goldberger A. L. (2009). Adaptive data analysis of complex fluctuations in physiologic time series. Adv. Adapt. Data Anal. 1 (1), 61–70. 10.1142/S1793536909000035 20041035 PMC2798133

[B54] Peng C. K. Havlin S. Stanley H. E. Goldberger A. L. (1995). Quantification of scaling exponents and crossover phenomena in nonstationary heartbeat time series. Chaos 5 (1), 82–87. 10.1063/1.166141 11538314

[B55] Pethick J. Casselton C. Winter S. L. Burnley M. (2021a). Ischemic preconditioning blunts loss of knee extensor torque complexity with fatigue. Med. Sci. Sports Exerc. 53, 306–315. 10.1249/MSS.0000000000002475 32735115 PMC7803438

[B56] Pethick J. Whiteaway K. Winter S. L. Burnley M. (2019c). Prolonged depression of knee-extensor torque complexity following eccentric exercise. Exp. Physiol. 104 (1), 100–111. 10.1113/EP087295 30485571

[B57] Pethick J. Winter S. L. Burnley M. (2021b). Fatigue-induced changes in knee-extensor torque complexity and muscle metabolic rate are dependent on joint angle. Eur. J. Appl. Physiol. 121 (11), 3117–3131. 10.1007/s00421-021-04779-1 34355267 PMC8505307

[B58] Pethick J. Winter S. L. Burnley M. (2018a). Caffeine ingestion attenuates fatigue-induced loss of muscle torque complexity. Med. Sci. Sports Exerc. 50 (2), 236–245. 10.1249/MSS.0000000000001441 28991045

[B59] Pethick J. Winter S. L. Burnley M. (2018b). Effects of ipsilateral and contralateral fatigue and muscle blood flow occlusion on the complexity of knee-extensor torque output in humans. Exp. Physiol. 103 (7), 956–967. 10.1113/EP086960 29719079

[B60] Pethick J. Winter S. L. Burnley M. (2019a). Fatigue reduces the complexity of knee extensor torque during fatiguing sustained isometric contractions. Eur. J. Sport Sci. 19 (10), 1349–1358. 10.1080/17461391.2019.1599450 30955469

[B61] Pethick J. Winter S. L. Burnley M. (2015). Fatigue reduces the complexity of knee extensor torque fluctuations during maximal and submaximal intermittent isometric contractions in man. J. Physiol. 593 (8), 2085–2096. 10.1113/jphysiol.2015.284380 25664928 PMC4405761

[B62] Pethick J. Winter S. L. Burnley M. (2016). Loss of knee extensor torque complexity during fatiguing isometric muscle contractions occurs exclusively above the critical torque. Am. J. Physiol. Regul. Integr. Comp. Physiol. 310 (11), R1144–R1153. 10.1152/ajpregu.00019.2016 27101290

[B63] Pethick J. Winter S. L. Burnley M. (2021c). Physiological complexity: Influence of ageing, disease and neuromuscular fatigue on muscle force and torque fluctuations. Exp. Physiol. 106 (10), 2046–2059. 10.1113/EP089711 34472160

[B64] Pethick J. Winter S. L. Burnley M. (2020). Physiological evidence that the critical torque is a phase transition, not a threshold. Med. Sci. Sports Exerc. 52 (11), 2390–2401. 10.1249/MSS.0000000000002389 32366801 PMC7556242

[B65] Pethick J. Winter S. L. Burnley M. (2019b). Relationship between muscle metabolic rate and muscle torque complexity during fatiguing intermittent isometric contractions in humans. Physiol. Rep. 7 (18), e14240. 10.14814/phy2.14240 31552708 PMC6759514

[B66] Pincus S. M. (1991). Approximate entropy as a measure of system complexity. Proc. Natl. Acad. Sci. U. S. A. 88 (6), 2297–2301. 10.1073/pnas.88.6.2297 11607165 PMC51218

[B67] Place N. Millet G. Y. (2020). Quantification of neuromuscular fatigue: What do we do wrong and why? Sports Med. 50 (3), 439–447. 10.1007/s40279-019-01203-9 31713783

[B68] Richman J. S. Moorman J. R. (2000). Physiological time-series analysis using approximate entropy and sample entropy. Am. J. Physiol. Heart Circ. Physiol. 278 (6 47-6), H2039–H2049. 10.1152/ajpheart.2000.278.6.h2039 10843903

[B69] Singh N. B. Arampatzis A. Duda G. Heller M. O. Taylor W. R. (2010). Effect of fatigue on force fluctuations in knee extensors in young adults. Philos. Trans. A Math. Phys. Eng. Sci. 368, 2783–2798. 10.1098/rsta.2010.0091 20439273

[B70] Skurvydas A. Brazaitis M. Kamandulis S. (2010). Prolonged muscle damage depends on force variability. Int. J. Sports Med. 31 (2), 77–81. 10.1055/s-0029-1241213 20221998

[B71] Slifkin A. B. Newell K. M. (1999). Noise, information transmission, and force variability. J. Exp. Psychol. Hum. Percept. Perform. 25 (3), 837–851. 10.1037/0096-1523.25.3.837 10385989

[B72] Slifkin A. B. Newell K. M. (2000). Variability and noise in continuous force production. J. Mot. Behav. 32 (2), 141–150. 10.1080/00222890009601366 11005945

[B73] Stergiou N. Harbourne R. T. Cavanaugh J. T. (2006). Optimal movement variability: A new theoretical perspective for neurologic physical therapy. J. Neurol. Phys. Ther. 30 (3), 120–129. 10.1097/01.NPT.0000281949.48193.d9 17029655

[B74] Stergiou N. (2004). Innovative analyses of human movement: Analytical tools for human movement research. Champaign: Human Kinetics.

[B75] Stergiou N. (2016). Nonlinear analysis for human movement variability. Florida, United States: CRC Press.

[B76] Taylor A. M. Christou E. A. Enoka R. M. (2003). Multiple features of motor-unit activity influence force fluctuations during isometric contractions. J. Neurophysiol. 90 (2), 1350–1361. 10.1152/jn.00056.2003 12702706

[B77] Taylor J. L. Gandevia S. C. (2008). A comparison of central aspects of fatigue in submaximal and maximal voluntary contractions. J. Appl. Physiol. 104 (2), 542–550. 10.1152/japplphysiol.01053.2007 18032577

[B78] Tyagi O. Zhu Y. Johnson C. Mehta R. K. Sasangohar F. Erraguntla M. (2020). Neural signatures of handgrip fatigue in type 1 diabetic men and women. Front. Hum. Neurosci. 14, 564969–565012. 10.3389/fnhum.2020.564969 33240061 PMC7680760

[B79] Vaillancourt D. E. Newell K. M. (2003). Aging and the time and frequency structure of force output variability. J. Appl. Physiol. 94 (3), 903–912. 10.1152/japplphysiol.00166.2002 12571125

[B80] Vassimon-Barroso V. Catai A. M. de Melo R. C. Quiterio R. J. Porta A. (2021). Complexity of knee extensor torque: Effect of aging and contraction intensity. J. Strength Cond. Res. 35 (34), 1050–1057. 10.1519/JSC.0000000000002888 30289867

[B81] Vázquez P. Hristovski R. Balagué N. (2016). The path to exhaustion: Time-variability properties of coordinative variables during continuous exercise. Front. Physiol. 7, 37–38. 10.3389/fphys.2016.00037 26913006 PMC4753307

[B82] Whiting P. Savovic J. Higgins J. P. T. Caldwell D. M. Reeves B. C. Shea B. (2016). Robis: A new tool to assess risk of bias in systematic reviews was developed. J. Clin. Epidemiol. 69, 225–234. 10.1016/j.jclinepi.2015.06.005 26092286 PMC4687950

[B83] Wolf A. Swift J. B. Swinney H. L. Vastano J. A. (1985). Determining Lyapunov exponents from a time series. Phys. D. Nonlinear Phenom. 16 (3), 285–317. 10.1016/0167-2789(85)90011-9

[B84] Yentes J. M. Hunt N. Schmid K. K. Kaipust J. P. McGrath D. Stergiou N. (2013). The appropriate use of approximate entropy and sample entropy with short data sets. Ann. Biomed. Eng. 41 (2), 349–365. 10.1007/s10439-012-0668-3 23064819 PMC6549512

[B85] Zwarts M. J. Bleijenberg G. van Engelen B. G. M. (2008). Clinical neurophysiology of fatigue. Clin. Neurophysiol. 119 (1), 2–10. 10.1016/j.clinph.2007.09.126 18039594

[B86] Zhu Y. Mehta R. K. Erraguntla M. Sasangohar F. Qaraqe K. (2020). Quantifying accelerometer-based tremor features of neuromuscular fatigue in healthy and diabetic adults. IEEE Sens. J. 20 (19), 11183–11190. 10.1109/JSEN.2020.2996372 PMC7729463 33287112

