# Supplementary material for: The use of non-linear tools to analyze the variability of force production as an index of fatigue: A systematic review
Source: Front Physiol. 2022 Dec 14;13:1074652. doi: 10.3389/fphys.2022.1074652 (PMC9795073; doi:10.3389/fphys.2022.1074652)
Supplement: Supplementary file 1 [file Table1.pdf]

Appendix 1

Table S1. Relevance assessment using PICO

Category	Targets Question	Review being assessed
Population (s):	Healthy adults.	It delivers. In some cases, also group with pathologies.
Intervention:	Exercise protocols with high force requirements that generate fatigue	It delivers. In different isometric and dynamic early stages.
Comparator (s):	Experimental group pre and post intervention	It delivers. Force, acceleration and motor analysis signals.
Outcomes (s):	Variability structure.	It delivers. Entropy measures, fractal measures, Lyapunov exponent and recurrence analysis.

Table S2. Quality Assessment Tool for Before-After (Pre-Post) Studies with No Control Group

	1. Was the study question or objective clearly stated?	2. Were eligibility/selection criteria for the study population prespecified and clearly described?	3. Were the participants in the study representative of those who would be eligible for the test/service/intervention in the general or clinical population of interest?	4. Were all eligible participants that met the prespecified entry criteria enrolled?	5. Was the s0061mple size sufficiently large to provide confidence in the findings?	6. Was the test/service/intervention clearly described and delivered consistently across the study population?	7. Were the outcome measures prespecified, clearly defined, valid, reliable, and assessed consistently across all study participants?	8. Were the people assessing the outcomes blinded to the participants' exposures/interventions?	9. Was the loss to follow-up after baseline 20% or less? Were those lost to follow-up accounted for in the analysis?	10. Did the statistical methods examine changes in outcome measures from before to after the intervention? Were statistical tests done that provided p values for the pre-to-post changes?	11. Were outcome measures of interest taken multiple times before the intervention and multiple times after the intervention (i.e., did they use an interrupted time-series design)?	12. If the intervention was conducted at a group level (e.g., a whole hospital, a community, etc.) did the statistical analysis take into account the use of individual-level data to determine effects at the group level?
Bastida-Castillo, Gómez - Carmona and Pino (2017)	Yes	Yes	Yes	NR	Yes	Yes	No	No	Yes	Yes	NR	NA
Bauer, et al. (2017)	Yes	Yes	Yes	NR	Yes	Yes	Yes	No	Yes	Yes	NR	NA
Chatain et al. (2020)	Yes	Yes	Yes	NR	Yes	Yes	Yes	No	Yes	Yes	NR	NA
Chatainet al. (2021).	Yes	Yes	Yes	NR	Yes	Yes	Yes	No	Yes	Yes	NR	NA
Cowley, Digwell and Gates (2014)	Yes	Yes	Yes	NR	Yes	Yes	Yes	No	Yes	Yes	NR	NA

Cruz-Montecinos, et al. (2018)	Yes	Yes	Yes	NR	Yes	Yes	Yes	No	Yes	Yes	NR	NA
Gates and Digwell (2008)	Yes	Yes	Yes	NR	Yes	Yes	Yes	No	Yes	Yes	NR	NA
Guzmán-Gónzalez, et al. (2020)	Yes	Yes	Yes	NR	Yes	Yes	Yes	No	Yes	Yes	NR	NA
Hollman, et al. (2020)	Yes	Yes	Yes	NR	Yes	Yes	Yes	No	Yes	Yes	NR	NA
Jiang, et al. (2019)	Yes	Yes	Yes	NR	Yes	Yes	Yes	No	Yes	Yes	NR	NA
Lin, Kuo and Hwang (2014)	Yes	Yes	Yes	NR	Yes	Yes	Yes	No	Yes	Yes	NR	NA
Oliveira et al. (2022)	Yes	Yes	Yes	NR	Yes	Yes	Yes	No	Yes	Yes	NR	NA
Pethick et al. (2020)	Yes	Yes	Yes	NR	Yes	Yes	Yes	No	Yes	Yes	NR	NA
Pethick et al.(2019)	Yes	Yes	Yes	NR	Yes	Yes	Yes	No	Yes	Yes	NR	NA
Pethick, Winter and Burnley (2016)	Yes	Yes	Yes	NR	Yes	Yes	Yes	No	Yes	Yes	NR	NA

Pethick, Winter and Burnley (2018a)	Yes	Yes	Yes	NR	Yes	Yes	Yes	No	Yes	Yes	NR	NA
Pethick, Winter and Burnley (2018b)	Yes	Yes	Yes	NR	Yes	Yes	Yes	No	Yes	Yes	NR	NA
Pethick, Winter and Burnley (2019a)	Yes	Yes	Yes	NR	Yes	Yes	Yes	No	Yes	Yes	NR	NA
Pethick, Winter and Burnley . (2015)	Yes	Yes	Yes	NR	Yes	Yes	Yes	No	Yes	Yes	NR	NA
Pethick, Winter and Burnley. (2019b)	Yes	Yes	Yes	NR	Yes	Yes	Yes	No	Yes	Yes	NR	NA
Pethick, Winter and Burnley. (2020)	Yes	Yes	Yes	NR	Yes	Yes	Yes	No	Yes	Yes	NR	NA

Pethick, Winter and Burnley. (2020)	Yes	Yes	Yes	NR	Yes	Yes	Yes	No	Yes	Yes	NR	NA
Tyagi, et al. (2020)	Yes	Yes	Yes	NR	Yes	Yes	Yes	Yes	Yes	Yes	NR	NA
Vazquez, Hristovski and Balagué (2016)	Yes	Yes	Yes	NR	No	Yes	Yes	No	Yes	Yes	NR	NA
Zhu et al. (2020)	Yes	Yes	Yes	NR	Yes	Yes	Yes	No	Yes	Yes	NR	NA

Note. NR = No reported; NA = No applicable

Table S3. Identifying concerns with the review process

**DOMAIN 1: STUDY ELIGIBILITY CRITERIA**		
**Signalling question**	**Rating**	**Reasoning**
1.1 Did the review adhere to pre-defined objectives and eligibility criteria?	Yes	The objectives and eligibility criteria were clearly defined before the review was launched.
1.2 Were the eligibility criteria appropriate for the review question?	Yes	The eligibility criteria were in line with the requirements of the objective.
1.3 Were eligibility criteria unambiguous?	Yes	Eligibility criteria were well defined to avoid ambiguity.
1.4 Were any of the restrictions in eligibility criteria based on the study characteristics appropriate (e.g. date, sample size, study quality, outcomes measured)?	Probably yes	We believe that the restrictions were appropriate based on the objective of the study.
1.5 Were any of the restrictions in eligibility criteria based on the sources of information appropriate (e.g. publication status or format, language, availability of data)?	Probably yes	We believe that the one restriction that was made is justified and will not affect the results obtained.
Concerns regarding specification of study eligibility criteria	Low	Most questions were answered with "Yes" or "Probably yes". Therefore, no potential problems were identified regarding the specification of the eligibility criteria. During the review an attempt was made to clearly specify the question and the objectives of the review, and to pre-specify and justify adequate and detailed eligibility criteria.

DOMAIN 2: IDENTIFICATION AND SELECTION OF STUDIES		
**Signalling question**	**Rating**	**Reasoning**
2.1 Did the search include an appropriate range of databases/electronic sources for published and unpublished reports?	Probably yes	Scopus, PubMed and SPORTDiscus (EBSCO) were included. This was judged to be an appropriate range.
2.2 Were methods additional to database searching used to identify relevant reports?	Yes	Additional searches were performed based on the list of references, articles and reviews included, and on the ResearchGate profiles of authors.
2.3 Were the terms and structure of the search strategy likely to retrieve as many eligible studies as possible?	Probably yes	We believe they were, and furthermore, in the event of any doubt in a study, it was studied in depth and it was decided whether or not to include it by consensus of all the authors.
2.4 Were restrictions based on date, publication format, or language appropriate?	Probably yes	There was only a language restriction, and we believe that this was not a relevant restriction.
2.5 Were efforts made to minimise error in the selection of studies?	Probably yes	The search was conducted by a single reviewer, but the inclusion decision was made by consensus of several reviewers. So, we think they probably were.
Concerns regarding methods used to identify and/or select studies	Low	The answer was "Yes" or "Probably yes", as the process was carefully designed to avoid the loss of relevant papers. We therefore believe that the risk of losing papers was low.

DOMAIN 3: DATA COLLECTION AND STUDY APPRAISAL		
**Signalling question**	**Rating**	**Reasoning**
3.1 Were efforts made to minimise error in data collection?	Probably no	Although the extracted data were reviewed by several authors, no detailed data review structure was followed.
3.2 Were sufficient study characteristics available for both review authors and readers to be able to interpret the results?	Yes	If they were available in the studies. In addition, the main characteristics are included in a table in the review.
3.3 Were all relevant study results collected for use in the synthesis?	Yes	The results of the pre- and post-differences were extracted to establish the effect of fatigue on the variability structure. This required finding out if there were significant differences between the pre- and post-moments.
3.4 Was risk of bias (or methodological quality) formally assessed using appropriate criteria?	Probably no	Given the characteristics of the studies we did not find a suitable tool to assess the risk of bias. Therefore, the reviewers' judgement was applied, but we cannot assure the validity of this method.
3.5 Were efforts made to minimise error in risk of bias assessment?	Probably yes	Yes, it was done through the assessment of one reviewer, and the checking of two reviewers.
Concerns regarding methods used to collect data and appraise studies	High	It is likely that by not conducting a risk of bias assessment with a validated tool there may be risk in this domain. However, given the objectives and knowledge of the topic, we believe that this risk may be acceptable.

DOMAIN 4: SYNTHESIS AND FINDINGS		
**Signalling question**	**Rating**	**Reasoning**
4.1 Did the synthesis include all the studies that it should?	Probably yes	All the articles that met the characteristics of the main study question were included in the review, for a total of 23 articles. However, for some of the secondary questions, not all studies could be included as they did not answer the secondary questions.
4.2 Were all pre-defined analyses reported or departures explained?	No	The review does not follow a published or accessible protocol.
4.3 Was the synthesis appropriate given the nature and similarity in the research questions, study designs and outcomes across included studies?	Probably yes	Although there was some heterogeneity in the studies because of the different measurement tools and variables used, they were grouped together to maximise the robustness of the synthesis. Given the main study question and the state of knowledge, we believe the synthesis was sufficiently robust.
4.4 Was between-study variation (heterogeneity) minimal or addressed in the synthesis?	No informatio n	As this was a systematic review in which the synthesis was done through narrative, these checks could not be applied.
4.5 Were the findings robust, e.g. as demonstrated through funnel plot or sensitivity analyses?	No informatio n	As this was a systematic review in which the synthesis was done through narrative, these checks could not be applied.
4.6 Were biases in primary studies minimal or addressed in the synthesis?	No	Although we consider that there is no significant bias in the constituent studies, as we have not assessed the risk of bias in the systematic review, we must indicate that there is a high risk of bias.
Concerns regarding the synthesis and findings	High	Although the findings were convincing, there are some limitations that have been noted and we cannot be sure that the risk is low.

Judging risk of bias

**RISK OF BIAS IN THE REVIEW**		
**Signalling question**	**Rating**	**Reasoning**
A. Did the interpretation of findings address all of the concerns identified the Phase 2 assessment?	Probably yes	Although some questions were answered negatively, we believe that the main research question can be answered quite solidly. Furthermore, the different limitations were pointed out in the limitations section.
B. Was the relevance of identified studies to the review's research question appropriately considered?	Yes	The included studies are directly applicable to the research question. Therefore, we believe they are likely to be used to draw robust conclusions.
C. Did the reviewers avoid emphasizing results on the basis of their statistical significance?	Probably yes	Given the number of results pointing in the same direction, we believe that this criterion was met in the main research question. In secondary questions in which the evidence was limited, no strong statements were made, or limitations were indicated.
Risk of bias in the review	Low	The concerns and limitations were adequately considered in the conclusions of the review. Therefore, the conclusions of the review are likely to be reliable.
